# Lipid droplets: platforms with multiple functions in cancer hallmarks

**DOI:** 10.1038/s41419-020-2297-3

**Published:** 2020-02-06

**Authors:** André L. S. Cruz, Ester de A. Barreto, Narayana P. B. Fazolini, João P. B. Viola, Patricia T. Bozza

**Affiliations:** 1 Laboratory of Immunopharmacology, Oswaldo Cruz Institute, FIOCRUZ, Rio de Janeiro, Brazil; 2 Laboratory of Physiopathology, Polo Novo Cavaleiros, Federal University of Rio De Janeiro (UFRJ), Macaé, Brazil; 3 Program of Immunology and Tumor Biology, Brazilian National Cancer Institute (INCA), Rio de Janeiro, Brazil

**Keywords:** Lipids, Cancer metabolism, Inflammation

## Abstract

Lipid droplets (also known as lipid bodies) are lipid-rich, cytoplasmic organelles that play important roles in cell signaling, lipid metabolism, membrane trafficking, and the production of inflammatory mediators. Lipid droplet biogenesis is a regulated process, and accumulation of these organelles within leukocytes, epithelial cells, hepatocytes, and other nonadipocyte cells is a frequently observed phenotype in several physiologic or pathogenic situations and is thoroughly described during inflammatory conditions. Moreover, in recent years, several studies have described an increase in intracellular lipid accumulation in different neoplastic processes, although it is not clear whether lipid droplet accumulation is directly involved in the establishment of these different types of malignancies. This review discusses current evidence related to the biogenesis, composition and functions of lipid droplets related to the hallmarks of cancer: inflammation, cell metabolism, increased proliferation, escape from cell death, and hypoxia. Moreover, the potential of lipid droplets as markers of disease and targets for novel anti-inflammatory and antineoplastic therapies will be discussed.

## Facts

- Lipid droplets are dynamic and multifunctional organelles involved in energy metabolism, signaling, and inflammatory mediator production.
- Lipid droplets accumulate in a variety of cancer cells.
- Lipid droplets modulate the cross-talk between tumors and other cell types in tumor microenvironment.
- Lipid droplet accumulation and catabolism are tightly coupled to energetic metabolism, cell signaling, and are critical to cancer cell proliferation, resistance to death, and aggressiveness.

## Open questions

- Does LD play a causal role or is a consequence of tumorigenesis?
- How important is LD accumulation during the distinct phases of tumor development?
- Can lipid droplet be a cytoplasmic hub for protumorigenic cellular signaling?
- Are there roles for inhibitors of lipid droplet biogenesis as target for cancer therapy?

## Introduction

Lipid droplets (LDs) are cytoplasmic lipid-enriched organelles delimited by a monolayer of phospholipid, which covers a hydrophobic core composed of neutral lipids, mainly triacylglycerol (TAG) and cholesteryl esters (CEs), with a diverse content of proteins that may vary according to the cell and stimulatory conditions. In addition, LDs are coated by structural proteins of the PAT family that include perilipin-1, perilipin-2, and perilipin-3^1–4^. LDs are organelles that originate and are intimately related to the endoplasmic reticulum (ER), formed by the transfer of lipids and proteins from the ER to newly formed LDs^5^. Although LD biogenesis involves specific and well-regulated mechanisms, the cellular and molecular mechanisms involved are still not completely understood.

**Fig. 1:**
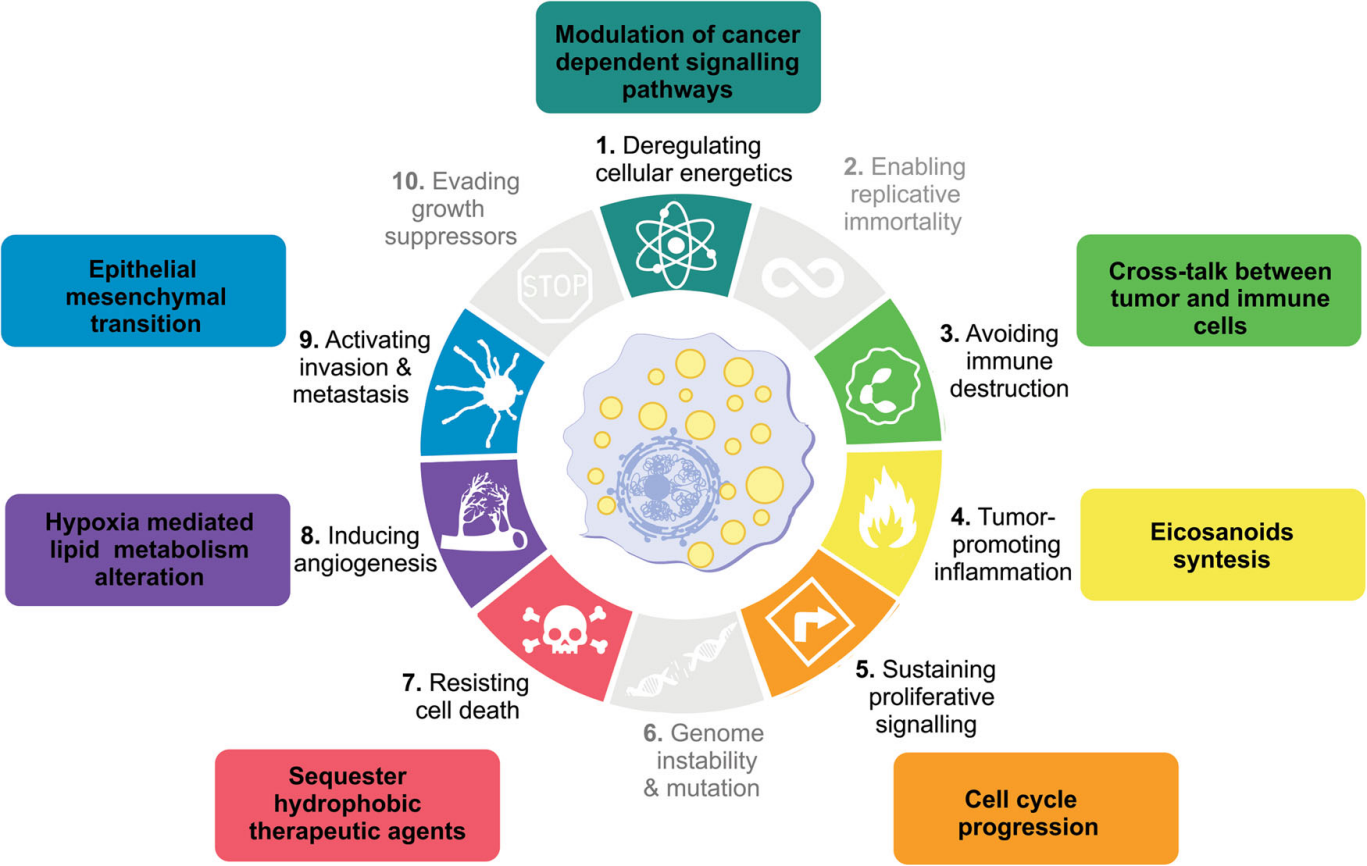
Lipid droplets as players in hallmarks of cancer. Based on the increasingly information about the role of lipid droplets in cancer, emerging from several different models, we suggest the association of lipid droplets with some of the currently established Hallmarks of Cancer—biological traits acquired by cells during cancer multistep development, a concept originally conceived by Hanahan and Weinberg in 2000. Although there are many unanswered questions of fundamental importance to better understand the relationship between these organelles and tumorigenesis, these associations may be explored for future anticancer therapies.

The first reports of LDs in human tumors date from the 1960s^6,7^; nevertheless, for many years, the study of LDs in cancer was restricted to descriptions in different tumors. A major breakthrough in LD research in cancer, came from the demonstration that LDs are major sites for prostaglandin E_2_ (PGE_2_) synthesis in colon cancer cells and have roles in tumor cell proliferation^8^. Over the last decade, there was a considerable expansion of the mechanisms that regulate the formation and functions of LDs in cancer. LDs have been identified in all the processes involved in cancer development, including initiation, promotion, and progression. Hanahan and Weinberg^9^ outlined hallmarks that characterized the capabilities acquired during tumor development. Among these are sustaining proliferation, resisting death, evading growth suppressors, promoting replicative immortality, activating invasion and metastasis, as well as promoting angiogenesis, genome instability, inflammation, energy metabolism deregulation, and evasion of immune destruction^10^. Here, we review the current knowledge of LD functions according to categories comprising the hallmarks of cancer, and two additional topics comprise LD roles in biomarkers and cancer stem cells (Fig. 1).

## Implications of lipid droplets in cancer

### Lipid droplets and metabolism in cancer

**Fig. 2:**
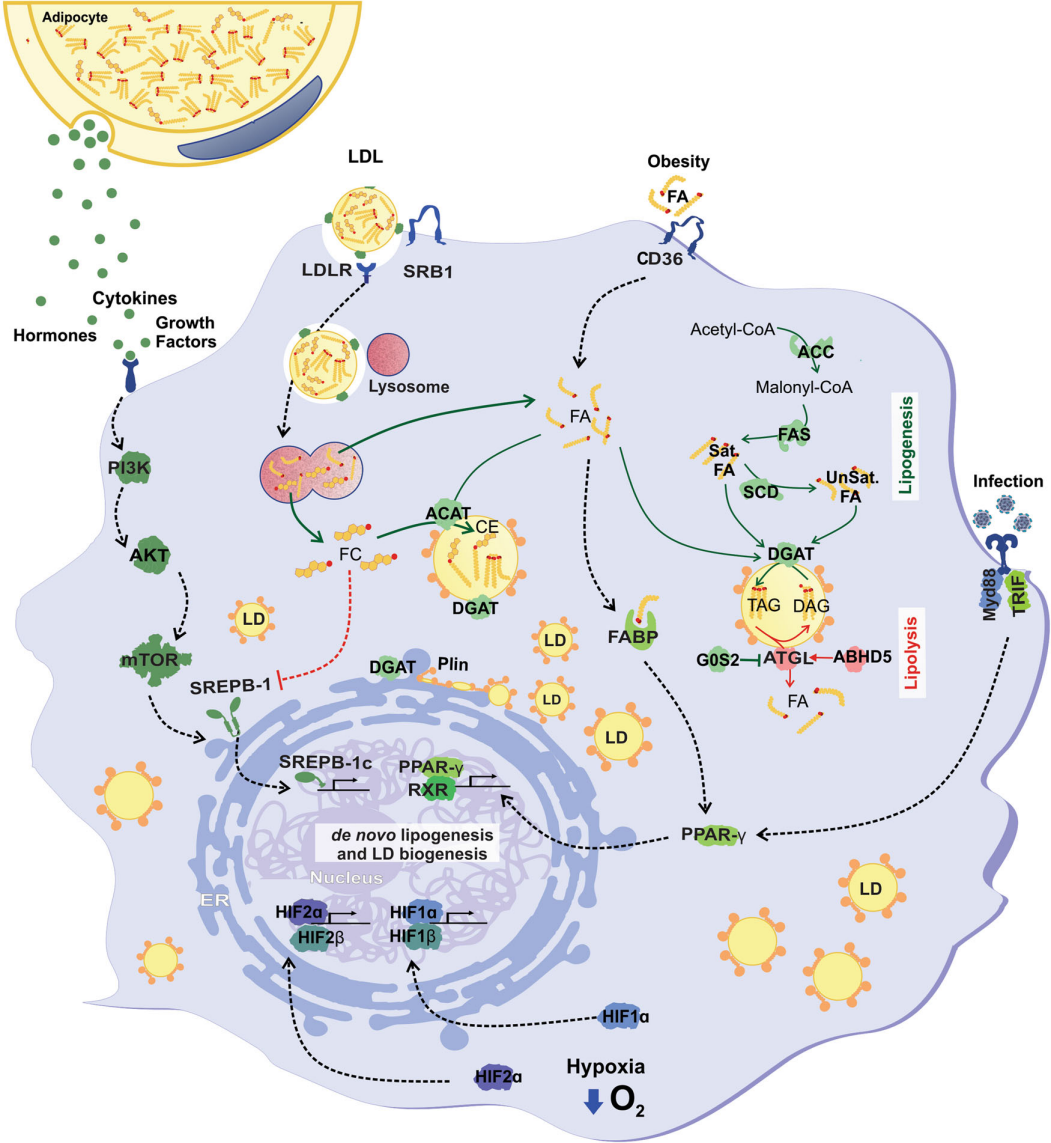
Mechanisms of lipid droplet biogenesis in cancer. Different stimuli and cellular pathways contribute for lipid droplets formation, depending on environmental conditions, such as hypoxia, obesity, infection, or extracellular signaling molecules. These processes invariably involve changes in gene expression that regulates de novo lipid synthesis, induction of extracellular lipid uptake and LD biogenesis. Lipid droplets formed by these different stimuli harbor specific lipid content; and a set of enzymes directly related to lipogenesis, such as FAS and DGAT, promote its increase. Lipolytic enzymes could also be located in lipid droplets for fatty acid mobilization upon activation. ABHD5 α-β hydrolase domain containing 5 (also known as CGI-58—Comparative Gene Identification-58), ACC acetyl-CoA carboxylase, ATGL adipose triglyceride lipase, DGAT diacylglycerol O-acyltransferase, DAG diacylglycerol, FA fatty acid, FABP fatty acid-binding protein, FAS fatty acid synthase, G0S2 G0/G1 switch 2, HIF hypoxia-inducible factors, mTOR mammalian target of rapamycin, PI3K phosphoinositide 3-kinase, PLIN2 perilipin-2, PPARγ peroxisome proliferator-activated receptor gamma, RXR retinoid X receptor, SatFA saturated fatty acid, SCD stearoyl-CoA desaturase, SREBP sterol regulatory element-binding protein, TAG triacylglycerol, TLR4 Toll-like receptor, TRIF TIR-domain-containing adapter-inducing interferon-β, UnsatFA unsaturated fatty acids, CD36 fatty acid translocase, CE cholesteryl ester, FC free cholesterol, LDL low-density lipoprotein, LDL-R low-density lipoprotein receptor, SRB1 scavenger receptor class B type, ACAT acyl-CoA:cholesterol acyltransferase.

Lipid droplet accumulation in transformed cancer cells involves complex and mediated mechanisms, including increased lipid uptake, de novo lipid synthesis and remodeling, as well as regulations in lipolysis (Fig. 2). Lipogenesis is paramount among the anabolic processes in cancer, where fatty acid (FA) synthesis is critical for generating building blocks for more complex lipids^11^. Signaling pathways that trigger tumor lipogenesis culminate in the accumulation of newly formed LDs^8,12–22^. Indeed, accumulating evidence support a relationship between tumor development and both lipolytic or lipogenic enzymes, implying that lipid mobilization from LDs may be an appropriate target for cancer therapy. The main factors in this phenomenon are sterol regulatory element-binding proteins (SREBPs), a key regulator of lipid homeostasis^23^, and mammalian target of rapamycin (mTOR), a crucial sensor that connects cellular growth to the availability of extracellular nutrients^24^. SREBP1 upregulation triggers tumor growth and LD accumulation together with lipogenesis enzyme overexpression^12,14,15,18,20,22,25,26^. Moreover, both mTOR catalytic complexes, namely, mTORC1 and mTORC2, were implicated in LD biogenesis in cancer by SREBP1-dependent^14,26^ and SREBP1-independent^20^ mechanisms.

Obesity and its associated metabolic dysregulation are established risk factors for many cancers. Obesity-drive ectopic LD accumulation in non-adipose tissues have been associated with insulin-resistance, cardiovascular disease, and cancer^1^. Different mechanisms may contribute to obesity-induced increased LDs in epithelial cells and other in non-adipose tissues. In addition to excess of free FAs and other lipids, adipose-derived leptin through mTOR activation may contribute to the obesity-related enhanced susceptibility to colon carcinoma by altering the intracellular lipid metabolism and inflammatory environment^27^.

TAG is one of the major neutral lipids in LDs. TAG is formed by a glycerol joined to three FAs and shares many steps with glycerophospholipid synthesis until the generation of DAG that is esterified by acyl-CoA diacylglycerol acyltransferase (DGAT) to TAG. The role of TAG metabolism remains poorly understood in cancer. Ackerman et al.^28^ showed that TAG acts as a buffer for lipid remodeling, especially under O_2_ and nutrient limitation. LD TAGs were enriched with unsaturated FAs, particularly oleate; however, during O_2_ and serum deprivation, oleate was released from LDs into phospholipid pools, which prevents cellular stress by avoiding the use of fully saturated, potentially toxic lipids^28^. They also showed that inhibition of both DGAT isoforms increased cancer cell death in vivo, impairing tumor growth^28^. In addition, DGAT1 was overexpressed in prostate cancer cells, and its inhibition decreased LD density, microtubule-organizing center numbers and microtubule stability, which affects cell migration and growth^29^.

TAG is sequentially hydrolyzed by three different lipases: adipose triglyceride lipase (ATGL), hormone sensitive lipase (HSL), and monoacylglycerol lipase (MAGL). Consecutively, they hydrolyze TAG, DAG, and MAG into a glycerol backbone and free FAs. ATGL function could be regulated by CGI-58/ABHD5, a coactivator, and G0S2 (G0/G1 switch gene2), an inhibitor^30^. The following data indicate that the role of lipases in cancer seems to be dependent on the cell type or protein studied. Loss of ATGL is a common feature in many human tumors and induces spontaneous lung cancer in animal models^31^. In addition, ATGL deletion can induce a more aggressive phenotype in lung cancer cells through lipid accumulation^32^. Moreover, CGI-58/ABHD5 deletion was correlated with cancer development and progression^33^ and epithelial–mesenchymal transition^33,34^. In contrast, ATGL inhibition by G0S2 decreased proliferation in tumor cell lines, suggesting that ATGL activity is common in some cancer types^35^. MAGL regulates the network of free FAs in tumors, such as colorectal cancer, neuroblastoma, hepatocarcinoma, and nasopharyngeal carcinoma, by enabling tumor cells to mobilize and utilize FAs from stored TAGs^36–40^. These released FAs, including lysophosphatidic acid, PGE_2_, and endocannabinoids, were involved in signal cascades that induce carcinogenesis, tumor progression, and metastasis^39,41^. Interestingly, lipolysis also plays an important role in cancer-associated cachexia, a multifactorial metabolic syndrome associated with loss of muscle and adipose mass^42^. Increased ATGL and, to a less extent, HSL activities were observed in white adipose tissue from cancer-associated cachexia patients, whereas ATGL-deficient mice with tumors maintained adipose tissue and gastrocnemious muscle mass^43^. Therefore, further studies are needed to elucidate the role of TAG lipases in cancer.

Another LD component is cholesteryl ester (CE), the storage form of cholesterol synthesized by acyl coenzyme A: cholesterol acyltransferase (ACAT)^44^. Free cholesterol maintains membrane fluidity and can be either de novo synthesized via the mevalonate pathway or taken up from exogenous lipoproteins^45^. Aberrant CE accumulation in LDs is an important target of tumor metabolism remodeling. Increased CE is a metabolic signature in renal cell carcinoma, glioblastoma, breast, prostate and pancreatic cancer^17,21,46–49^. In addition, CE accumulation was positively correlated with advanced clinical staging, metastasis, and poor survival^21,46–48^. Inhibition of ACAT significantly suppressed cancer proliferation, migration, invasion, and tumor growth in vitro and in vivo^17,21,46^. CE accumulation is driven by PTEN loss and PI3k/Akt/mTOR upregulation^21^ and modulates signaling pathways, such as SREBP by blocking the SREBP-negative feedback loop caused by excess free cholesterol, maintaining tumor growth^17,21,46^, and also Wnt/β-catenin by regulating FA availability for Wnt3a acylation^50^. These studies suggest that cholesterol esterification may be a major target in cancer therapy.

LDs are directed to autophagy pathways as an alternative route for lipid storage mobilization^51,52^. Lipid droplet inclusion in autophagossomes and subsequent degradation in lysosomes (known as macrolipophagy) regulates cellular lipid content, as inhibition of lipophagy decreases TAG breakdown^53^. Upon engulfment, LD content are broken by lysosomal acid lipases (LAL), mostly known for its deficiency in Wolman disease and CE storage disease^54,55^. Another mechanism for LD hydrolysis in lysosomes is through chaperone-mediated autophagy (CMA), a lysosomal proteolysis carried by heat shock protein 70 (HSP70)^56^ and lysosome-associated membrane protein 2A (LAMP-2A)^57^. Degradation of perilipin-2 and perilipin-3 by CMA act as a prerequisite to stimulate both ATGL lipolysis and macrolypophagy^58^. Several CMA-targeted proteins are relevant to cancer biology^59^, though the relationship between CMA-dependent lipolysis and cancer is currently unknown. For more in-depth information, we recommend recently published reviews where lipophagy regulatory mechanisms and functions in other physiopathological conditions are comprehensively discussed^52,60^.

Though poorly studied, lipophagy appears to play a dual role in cancer. LDs degradation by lipophagy increases viability of hepatocarcimoma cells during starvation^61^, and protects androgen-sensitive prostate cancer cells during androgen-deprivation in vitro^62^. Also, pharmacological inhibition of lipolysis in colorectal cancer cell line induced both LDs accumulation and cell death, while altering the profile of cancer stem cells toward a more invasive mesenchymal phenotype^63^. On the other hand, lipophagy can display an antitumoral role in some cancer models. Overexpression of autophagy regulatory protein ATG14 increased LD lipophagy while sensitized HeLa cells to apoptosis^64^.

### Lipid droplets in inflammation and avoiding immune destruction

In inflammation and cancer, LDs are linked to the regulation of immune and inflammatory responses by acting as specialized hubs of signaling with major roles in eicosanoid and other lipid mediator formation^65^. Eicosanoids are signaling molecules derived from the enzymatic oxygenation of arachidonic acid (AA), which control key cellular processes, including cell activation, metabolism, proliferation, and death^66^. It is well established that LDs are sites of esterified AA as well as of several enzymes involved in eicosanoid synthesis including cPLA_2_, cyclooxygenases and prostaglandin synthases^8,67–71^. By means of eicosacell, a technique to localize newly formed eicosanoids at their sites of synthesis^72^, it was established that LDs are major intracellular locales for the activation-elicited formation of PGE_2_ in cancer cells^8^. Moreover, leukocytes, endothelial and epithelial cells involved in pathological conditions, such as in cancer, hypoxia, and during infections were shown to contain increased numbers of eicosanoid-synthesizing LDs leading to amplified eicosanoid production^8,73–76^.

**Fig. 3:**
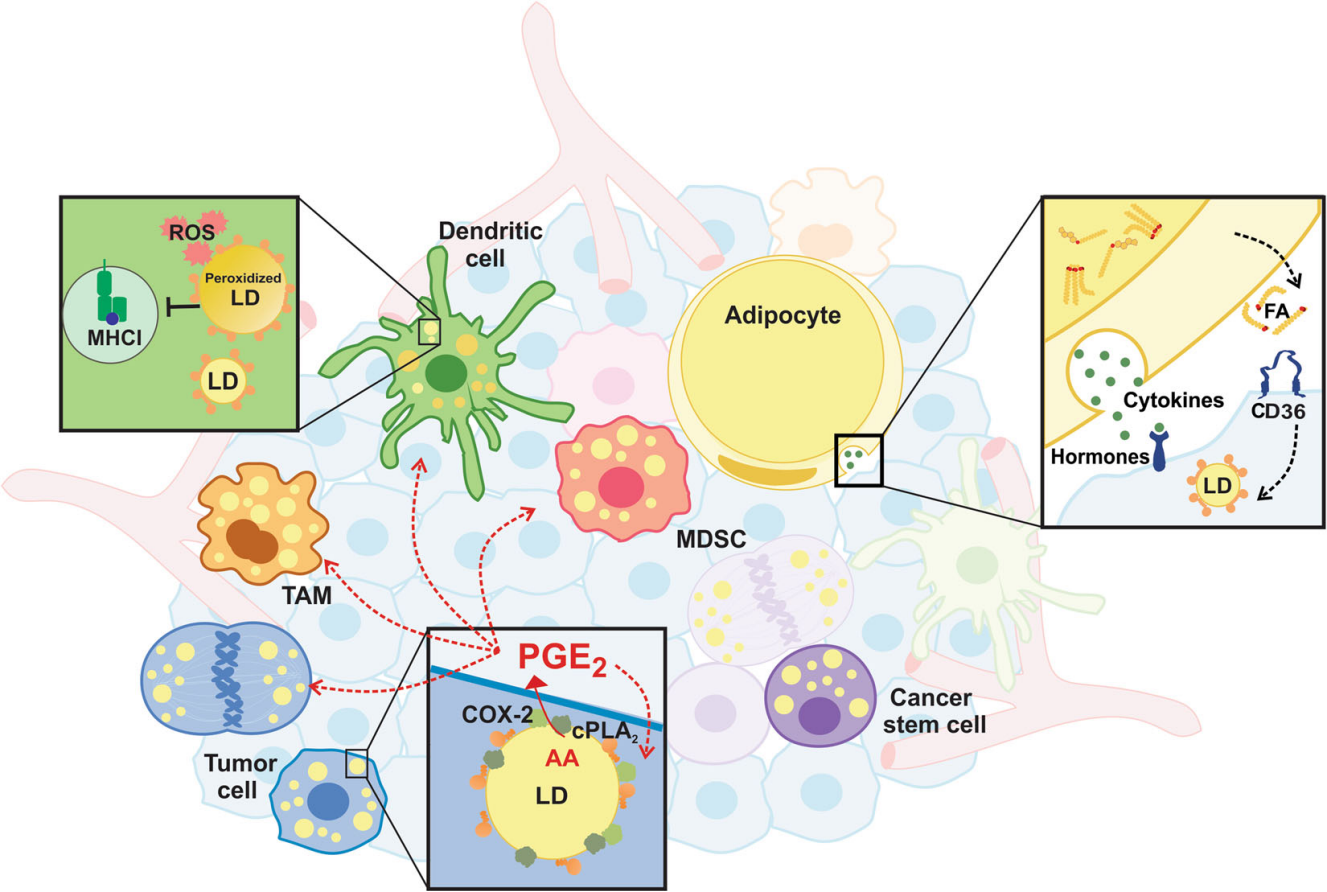
Lipid droplets roles in tumor microenvironment. Lipid droplets (LDs) were associated with distinct roles in heterogeneous cell population of tumor microenvironment. In tumor cells, LDs are sites of PGE_2_ (prostaglandin E_2_) synthesis, an important immune suppressive eicosanoid, and are associated with proliferation and activation of cancer stemness pathways. Adipocytes release cytokines and fatty acids to fuel metastasis and aggressiveness. In myeloids derived cells, LDs were associated with polarization of TAM (tumor-associated macrophage), a modulatory phenotype of MDSCs (myeloid-derived suppressor cells) and in dendritic cell, LDs enriched with oxidized triacylglycerol species were associated with antigen presentation dysfunction. ROS reactive oxygen species, MHCI major histocompatibility complex class I protein, COX-2 cyclooxygenase-2, cPLA_2_ cytosolic phospholipase A2, AA arachidonic acid, FA fatty acid, CD36 fatty acid translocase.

The amplification of eicosanoid formation through the compartmentalization of eicosanoid-synthetic machinery at LDs in tumor cells may have implications to promote tumor growth by paracrinally regulating cancer cells, as well as by orchestrating the complex interactions to establish the tumor microenvironment (Fig. 3). Indeed, LD and LD-derived PGE_2_ were shown to promote tumor epithelial cell proliferation^8,27,73^. Also, several reports indicate that PGE_2_ has a protumoral role by stimulating tumor cell proliferation and suppressing host immune surveillance of tumor^77–81^.

The tumor microenvironment (TME) is a term that refers to a heterogeneous cell population of cancer cells and host resident and recruited cells, secreted factors and extracellular matrix that constitute the tumor mass^82^. Furthermore, the TME plays a critical role in the prognosis of tumor development and therapeutic responses^83^. Recent studies indicate that LDs modulate the cross-talk between tumors and phenotypic modulation of immune cells, mainly myeloid cells, such as tumor-associated macrophages (TAMs), myeloid-derived suppressor cells (MDSCs), and dendritic cells (DCs)^84–89^ (Fig. 3). Indeed, as mentioned above, LDs are involved in increased eicosanoid synthesis in tumor cell LDs and inflammatory cells^8,71,90^, suggesting roles of LDs as an eicosanoid production site in modulating TME cells. In addition to eicosanoids synthesis, LDs are emerging as sites for regulation of different signaling pathways with potential functions in TME regulation.

TAMs are the major tumor-infiltrating leukocytes and promote malignant progression by stimulating angiogenesis and tissue remodeling and preventing immune destruction^91^. The role of LDs in these cells are still not fully established, since the literature demonstrates both protumorigenic and antitumorigenic functions. FA-binding protein E (E-FABP), a lipid-binding chaperone, is highly expressed in TAMs presenting anticancer activities^84^. In this scenario, IFN-β signaling is regulated through E-FABP-mediated LD biogenesis, recruiting natural killer cells to the tumor stroma^84^. In contrast, cancer cells stimulate TAM differentiation toward a protumorigenic phenotype by a caspase-1-dependent, nonconventional cleavage of PPARγ^87^. Cleaved PPARγ then translocates to mitochondria and reduces FA oxidation, increasing LDs and promoting protumor TAM differentiation^87^. Also, Wu et al.^92^ showed that unsaturated FA, oleate, polarizes bone marrow-derived myeloid cells into an immunosuppressive TAM. This phenotype is dependent on mTOR signaling, LD formation, and utilization by mitochondrial respiration. Moreover, LDs from TAMs were shown as an effective target to avoid TAM polarization and tumor growth^92^. Further work to elucidate the functions of the LDs in TAM differentiation and the origin of its lipids are necessary.

MDSCs are a diverse group of immune cells from the myeloid lineage with strong immunoregulatory properties^93^, which are shown to depend on both oxidative stress and lipid metabolism^94–96^. Tumor-infiltrating MDSCs increase FA uptake and oxidation, mitochondrial mass, and oxygen consumption rate. The inhibition of FA oxidation blocks the immunosuppressive functions of MDSCs, enhancing the efficacy of cancer immune therapy^94^. These findings encouraged other studies focused on the role of lipids in the induction of the regulatory phenotype of MDSCs^86,88^. Wu et al.^88^ demonstrated that unsaturated FAs, but not saturated FAs, are capable of inducing a modulatory phenotype in these cells paralleled by increased LD formation. The inhibition of LD formation by DGAT blockade abrogated the MDSC phenotype, while the inhibition of *de novo* FA synthesis had no effect, suggesting a critical role for exogenous FA and LD biogenesis. In addition, Al-Khami et al.^86^ reached similar conclusions when evaluating a tumor-bearing mouse model. They observed that the tumor-released cytokines G-CSF and GM-CSF triggered lipid influx and LD biogenesis, oxidative metabolism and T-cell suppression. They verified that exogenous lipoproteins and unsaturated FAs, but not saturated FAs, enhanced the generation of immunosuppressive MDSCs. These results showed that the LD biogenesis necessary to regulate phenotype MDSCs in cancer was triggered by exogenous lipids. Although the source of lipids in the TME was not evaluated, the specific induction by unsaturated FAs may provide clues about the mechanisms similar to that of the DC regulation described below.

DCs are central in the anticancer response due to cross-presentation of tumor-associated antigens via MHC-I complexes to CD8^+^ cytotoxic T cells^93^. Although the presence of DCs is associated with a better prognosis, studies in tumor-bearing mice showed impaired cross-presentation by DCs in the TME^97–100^. There are conflicting data on the role of LDs, which are associated with both promotion and inhibition of cross-presentation in tumor-infiltrating DCs^85,101–103^. These differences may be caused by LD quality, not quantity, and related to DC antigen presentation dysfunction^103^. Veglia et al.^89^ showed that LDs from tumor-infiltrating DCs are enriched with oxidized triacylglycerol species. In addition, oxidized LDs sequestrated HSP70, which directed pMHCI localization to lysosomes rather than to the plasma membrane^89^. Though the authors did not confirm the TME lipid source, it would reasonable to suggest the involvement of cancer lipogenesis. Thereafter, Jiang et al. confirmed that FASN overexpression of tumor cells was responsible for elevated levels of LDs and subsequent inhibition of DCs in an ovarian cancer mouse model^104^. FASN silencing in cancer cells decreases LDs in DCs, consequently increasing infiltrative T cells and delaying tumor growth, which suggests that tumor cell lipogenesis could be involved in anticancer immunity^104^.

In conclusion, these data demonstrate that LDs are associated with the immunometabolic modulation phenotype of myeloid cells, which largely culminate in cancer immune evasion. However, more research is necessary to understand the exact mechanisms of how LDs are involved in phenotype modulation^89^. In the DC studies, the combination of a lipid-enriched microenvironment and oxidative stress was necessary to trigger modulation. High levels of circulating lipids and oxidative stress are widely described in many tumors and are associated with a poor prognosis^105–107^. The identification of the lipid source used in LD biogenesis may also be an important aspect in the signaling in which these organelles are involved, since these lipids may come from both external sources, such as tumor cells and adipose tissue, and from intracellular sources, such as de novo synthesis or autophagy. Surprisingly, cell free LDs were described in a 3D bioengineered brain tumor glioblastoma tissue platform, where it was suggested may participate in drug response, however, the role and mechanism remain unclear^108^. In addition, it is necessary to determine how LDs are involved in the exclusion of T cells from the TME, since this may be an intriguing target in immune cancer therapy.

### Lipid droplets in cell proliferation

Accumulating evidence have shown that an increase in LD numbers occurs in cells undergoing proliferation, which is a common feature in many neoplastic processes, suggesting LD may contribute to cell proliferation^109^. Although no definitive studies establish a causal link between the increase in LD numbers and cancer development, recent studies are starting to shed light in this process. Indeed, emerging data associates increased LD biosynthesis and cell cycle progression. It was recently described that cell cycle progression regulates the number and cellular localization of LDs in nontransformed cells, with an increase in LDs numbers and dispersed subcellular localization upon entering S phase^110^. Moreover, detailed analysis of the distribution of lipid droplets during mitosis showed their polarization before cell division^110^. In addition, it was observed that the yeast lipase Tgl4 (human ATGL analog) is a target for phosphorylation by the major cell cycle regulator Cdc28 (human CDK1 analog), which is necessary for Tgl4 activity and cell cycle progression^111^. In mammals, a lipid-mediated PTEN-dependent late G_1_ checkpoint was recently described^112^. In this work, lipid deprivation in culture media was able to induce G_1_ arrest in several cancer cells, except in clear-cell renal carcinoma cells, where it is suggested that an increase in LDs contributes to bypass this checkpoint^112^. Collectively, these results suggest that cell cycle progression and lipid homeostasis are coordinated by a shared mechanism acting at the G_1_/S transition, thus suggesting that lipid droplet maintenance, biogenesis, or consumption is involved in cell cycle progression through S phase.

Activation of specific signaling pathways in colon cancer cells is linked with LD formation and cell growth modulation^8^. FOXO3 plays a pivotal role in inhibiting colon cancer cell proliferation, mainly through upregulation of the cell cycle inhibitor p27kip1^113^. However, FOXO3 activity is dependent on LD density, and an increase in LD numbers induced the loss of FOXO3 and p27kip1 expression^114^. Increased LD density promoted proliferation of colon cancer cells in a FOXO3 loss-dependent manner^114^. In addition, the mouse model for FOXO3 deficiency resulted in a decrease in Sirtuin6, a negative regulator of lipid metabolism, revealing the existence of a regulatory network between LD biogenesis and FOXO3 activity^114^ Interestingly, in vitro cellular transformation using H-RasV12 oncoprotein was associated with LD accumulation^8,110^ and an increase in perilipin-2 protein levels^110^, although perilipin-2 overexpression alone was not enough to induce cell transformation in murine fibroblasts^110^.

Of note, a variety of signaling-associated proteins have been found within LDs, suggesting a key role for this organelle as a cytoplasmic hub favoring quick proliferative signaling. Proteins with well-established roles in oncogenic transformation, tumorigenesis, and metastasis, including PI3K, ERK1, ERK2, p38, PKC, and caveolin, were shown to localize to LDs in a variety of cell types^69,115,116^. Nevertheless, until now, no study has unraveled the actual role of lipid droplet-resident kinases in cell proliferation. Hence, LDs could be a potential downstream target against uncontrolled triggering of the membrane receptor signaling cascade. Despite all suggestive data linking LDs and cell cycle progression, further studies are necessary to characterize whether LDs play a direct role in cell proliferation.

### Lipid droplets in apoptosis and cell death

It has been reported that apoptosis induction leads to an early onset and subsequent accumulation of LDs^117–119^. Indeed, LD formation during apoptosis may delay the accumulation of toxic FAs^120,121^. Apoptosis-induced activation of p53 and inhibition of mTOR^122^ and MYC^123^ in tumor cells leads to lipid accumulation due to inhibition of FA β-oxidation and redirection of FAs to de novo lipogenesis. In addition, the increase in LD content has been used as an in vivo marker of post treatment tumor cell death through 1H nuclear magnetic resonance spectroscopy, a noninvasive diagnostic technique to detect the earliest signs of cell death following cancer treatment^119,124^.

Increased LDs in cancer cells may play an indirect role in maintaining cell survival during cancer therapy. An increase in LD numbers was previously observed in drug-resistant cancer cells, and thus, these organelles were postulated to sequester hydrophobic therapeutic agents, reducing drug effectiveness^125,126^. The development of drug-resistant cells derived from myeloid leukemia also revealed a positive correlation between increased LD content and resistance to an aminopeptidase inhibitor, along with activation of the ERK/Akt/mTOR survival pathway^127^. These data prompt the idea of hampering LD biogenesis to improve cancer therapy efficiency. Interestingly, reduction of LD formation by inhibition of cPLA2α enhanced the effectiveness of the anticancer agent curcumin in glioblastoma cells^128^. Hence, impairing LD drug sequestration could be interesting in a variety of other multidrug resistance scenarios to improve cell death upon antineoplastic drug administration.

Increased neutral lipids may enable further survival of cancer cells through other mechanisms. A recent study described a protective role of LDs in colorectal cancer cells against chemotherapy-induced cell death. LD biogenesis mediated by lysophosphatidylcholine acyltransferase 2 (LPCAT2) in these cells impaired ER stress pathways, resulting in diminished calreticulin (CRT) membrane exposure and, consequently, a reduction in immunogenic cell death after treatment^129^. Cell-surface CRT exposure is a key feature in anticancer immune responses^130^, and interestingly, CRT appeared to be sequestrated in LDs in these chemoresistant colorectal cells^129^. Other studies also revealed altered lipid metabolism in other resistant cancer cell lines, such as breast^125,131,132^ and ovarian cancer^133^; thus, LD formation may be involved in drug resistance. An interesting example was recently described in hypoxic conditions, where increased expression of acylglycerol-3-phosphate acyltransferase 2 (AGPAT2) was directly involved in LD accumulation and cell survival upon etoposide treatment in different cancer cell lines^134^.

### Lipid droplets in hypoxia and angiogenesis

Hypoxia is defined as a reduction of oxygen (O_2_) concentration, a condition encountered in a variety of pathological conditions. Hypoxia responses are transcriptionally regulated by the hypoxia-inducible factor (HIF) family, which includes heterodimeric transcription factors consisting of an oxygen-regulated α-subunit (HIF-1α or HIF-2α) and a constitutively expressed β-subunit (HIF-β/ARNT)^135–137^. Hanahan and Weinberg suggest that the hypoxia response system not only causes an induction of the “angiogenic switch” in cancer^138^, but is also one of the factors that acts in the reprogramming of cancer cell metabolism^10,139^. In fact, evidence points out important changes in lipid metabolism in these instances. Increased FA synthesis is thought to be stimulated in low O_2_ conditions; for example, FA synthase is upregulated during hypoxia through the HIF-1α/Akt/SREBP-1 signaling pathway^140^, whereas HIF-1α activation increases glycolysis and free FA uptake by upregulating PPARγ expression^141^. This supports the idea that hypoxia responses can significantly alter cell lipid metabolism in several pathologies, including cancer, and that LDs would accumulate in hypoxic cells. Indeed, an inverse correlation between the oxygen concentration and LD levels was first observed in endothelial cells derived from bovine aortic or pulmonary vascular beds, emerging as a specific rather than a standardized response for any variety of cellular stresses^142^. Accordingly, perilipin-2 expression was increased in cancer cell lines under hypoxic conditions, as well as in the liver of mice treated with CoCl_2_, an inducer of hypoxia-like responses^143^. LD staining was also found in highly hypoxic cells located at the periphery of necrotic areas of intracerebral glioma^144^. In this scenario, HIF transcription factors seem to portray specific roles in LD regulation. Interestingly, HIF-2α—but not HIF-1α—promotes *PLIN2* gene overexpression in clear-cell renal cell carcinoma, and perilipin-2 accumulation in these cells was associated with increased cancer cell viability^145^. Likewise, expression of both HIF-1α and HIF-2α was found to be important for establishing an invasive and metastatic phenotype in triple-negative breast cancer cells^146^, but single inhibition of HIF-2α expression alone resulted in altered metabolism and reduced formation of LDs in these cells^146^. In addition, in a mouse glioblastoma xenograft model, hypoxia induced LD accumulation in an HIF-1α-dependent manner due to increased FA uptake but not de novo lipid synthesis^147^. FABP3, FABP7, and perilipin-2 were essential for the formation of LDs under hypoxic conditions in this work^147^, and disruption of FABP3, FABP7, or perilipin-2 expression in this model reduced ATP production and increased ROS levels, which were accompanied by a decrease in cell growth and survival both in vitro and in vivo^147^. This evidence highlights the importance of lipid modulation during hypoxia as an important mechanism that directly regulates cell survival and aggressiveness.

### Lipid droplets in cancer aggressiveness, invasion, and metastasis

Epithelial–mesenchymal transition (EMT) is the first of several steps toward a carcinoma metastasis event. In this process, carcinoma cells lose epithelial traits, such as apical–basal polarity and epithelial cell junctions, and display mesenchymal cell morphology and increased cell migration potential^148^. It was recently shown that the lipid profile differs between epithelial and mesenchymal breast cancer cells, revealing that monounsaturated lipids and de novo FA synthesis are markedly characteristic of epithelial cells, whereas reduced lipogenesis, increased polyunsaturated FA levels, and the expression of genes involved in TAG synthesis and LD formation were mainly traits of mesenchymal breast cancer cells^149^. In parallel, loss of CGI-58/ABHD5 promotes invasion and proliferation in prostate cancer through an ATGL-independent mechanism and correlated with increased aerobic glycolysis and loss of E-cadherin expression and Snail accumulation, markers of the EMT process^34^. However, the importance of LD accumulation in cell invasion and metastasis is still debatable, particularly why and when lipid mobilization from LDs would be necessary to trigger a more aggressive phenotype in cancer cells. Wright and collaborators discuss the necessity of an LD increase in primary cancer cells prior to a metastasis event. In these observations, in vitro accumulation of prometastatic protein CDCP-1 (CUB-domain containing protein 1) decreased the lipid content in triple-negative breast cancer cells and was correlated with augmented invasion in 3D culture^150^. Interestingly, primary tumors in vivo displayed increased LDs, along with decreased CDCP-1 activity, when compared with their corresponding metastatic nodules^150^.

Some studies also indicate that fat mobilization between stromal and cancer cells is required for metastasis and cancer aggressiveness. Metastasis of ovarian cancer to the omentum was shown to be mediated by local adipocytes that released cytokines and provided FAs to cancer cells, which displayed increased LD formation and β-oxidation, a mechanism dependent on FABP4^125^. Adipocyte-induced FA translocator CD36 expression confers a more aggressive phenotype in ovarian cancer cells. In addition, CD36 inhibition was sufficient to reduce LD accumulation in cocultured cancer cells and limit tumor growth and invasion both in vitro and in vivo^151^. Indeed, CD36^+^ cells were previously highlighted as initiators of metastasis in mouse oral squamous cell carcinomas, and CD36 expression correlated drastically with poor prognosis in lung squamous cell cancer, bladder cancer, or luminal A breast cancer^152^. Moreover, ATGL accumulation and increased activity were observed predominately in aggressive breast cancer cell lines, and its expression increased in cells with direct contact with adipocytes in primary human breast cancer samples^153^. These findings add a new layer of complexity to the implications of the TME in cancer aggressiveness.

## Lipid droplets and cancer stem cells

Cancer stem cells (CSCs) comprise a small subpopulation of malignant cells that can propagate clones indefinitely as a self-renewal feature and also maintain the tumor by generating heterogeneous cancer cells that compose the bulk of the tumor mass^154,155^. Consequently, a single CSC holds an inherent potential for cancer initiation^155–157^ and is directly involved in cancer therapy resistance^158^ and efficiency^155^. The identification and isolation of CSCs from different cancer types are still matters of discussion^155,159–161^, and currently, the idea that stemness is a flexible, reversible trait of some cancer cells is being upheld^160^.

Current data show that CSCs have higher LD contents than differentiated tumor cells^162–164^. In a colorectal cancer model, cells with a high LD content showed CSC tumorigenic features in vitro and in vivo. In addition, there was a positive correlation between a high LD content, CD133 expression, and Wnt/β-catenin upregulation^162^. In the ovarian cancer cell population, the activity of stearoyl-CoA desaturase 1 (SCD1), an enzyme involved in monounsaturated FA synthesis, was strongly associated with LD levels and cancer stemness^163^. The ovarian CSC population had higher levels of LDs and unsaturated FAs. SCD1 blockade decreased LDs and impaired cancer stemness by inactivating the NF-кB pathway^163^. In addition, some reported that CSCs accumulate LDs to use as lipid reserves for energy supply^164,165^. Singh et al.^165^ showed that blocking lipolysis by targeting vesicle-mediated COPI complex, which transports lipases to the LD surface, starves CSCs to death. In addition, a CSC glioblastoma population showed a strong dependence on oxidative metabolism, FA uptake and high LD content, which is preferentially metabolized under glucose-deprived conditions^164,166,167^. In summary, the role of LDs in CSCs was associated with both energy demands and activation of cancer stemness pathways, such as Wnt/β-catenin and NF-κB signaling^162–165^. These data demonstrate that LDs are important for CSC maintenance, but further studies are needed to clarify their role and possible application as CSC-targeted therapy.

## Lipid droplets as a cancer biomarker

From the first observations in the late 1960s^6,7^, the indication of the increased LD numbers in cancer has raised the possibility of using the detection of LDs as biomarkers for diagnosis and prognosis (Table 1). Most studies correlate PAT proteins expression, mostly perilipin-2, with clinical–pathological features, since RNA from tumor samples or paraffin-embedded tissues is a readily available resource in biomarker studies. Overexpression of PAT proteins has been correlated with the differentiation between malignant and benign tissues^168–172^, clinical staging^171,172^, invasion^171–174^, and survival in several tumors^172,174–176^.

**Table 1:** Tumors where altered lipid droplets or expression of lipid droplet-associated proteins is observed.

Tissue	Tumor type	PLINs expression^a^	Lipid droplets	References
Brain	Human brain tumor	ND	+	^190^
	Glioma	High PLIN3	+	^46^
		ND	+	^191^
Breast/mammary gland	Apocrine carcinoma	Low PLIN3	ND	^192^
		High PLIN2	+	^193^
		High PLIN2	ND	^194^
	Carcinoma of the breast	ND	+	^195^
		ND	ND	^6,47^
	Invasive ductal carcinoma	Low PLIN2	−	^192^
		PLIN3^b^	−	^196^
	Invasive lobular carcinoma	Low PLIN2	−	^192^
Cervix	Cervical dysplasia	High PLIN3	−	^173,197^
	Invasive carcinoma	High PLIN3	−	^173,197^
Colon	Colon Adenocarcinoma	High PLIN2	+	^8,188^
		High PLIN2	ND	^110^
		High PLIN2, PLIN3	+	^192^
		PLIN2	ND	^187^
	Hyperplastic Polyps	High PLIN2	+	^198^
		PLIN2	ND	^183^
	Colorectal Cancer	PLIN2	+	^92^
Esophagus	Esophageal adenocarcinoma	PLIN2	ND	^185^
Head and Neck	Mammary analog secretory carcinoma	PLIN2	+	^199^
	Sebaceous carcinoma of the tongue	High PLIN2	+	^200^
Kidney	Clear-cell renal carcinoma	PLIN1, PLIN2, PLIN3	+	^192^
		High PLIN2^b^	−	^175,201^
		High PLIN2^b^	ND	^176^
		High PLIN2^b^	+	^13^
		High PLIN2	+	^202^
		High PLIN2^b^, PLIN1, PLIN3	+	^203^
		High PLIN3^b^	+	^172^
		ND	+	^49^
Larynx	Laryngeal squamous cell carcinoma	PLIN2, PLIN3, PLIN1	−	^192^
Liver	Cholangiocarcinoma	High PLIN2, low PLIN1	NC	^192^
	Hepatocellular carcinoma	High PLIN2, low PLIN1	+	^192^
		High PLIN2^b^	−	^204^
		ND	+	^205^
	Clear-cell hepatocarcinoma	ND	+	^206,207^
	Adrenal rest tumor	ND	+	^208^
Lung	Large cell lung carcinoma	High PLIN2, PLIN3	+	^192^
	Lung adenocarcinoma	High PLIN3	+	^192^
		High PLIN2	ND	^168^
		High PLIN2	+	^174^
	Lung squamous cell carcinoma	High PLIN2, PLIN3	+	^192^
	Sarcomatoid/pleomorphic lung carcinoma	PLIN2	ND	^168^
		Low PLIN2, high PLIN3	−	^192^
Lymphoma	Burkitt lymphoma	PLIN2^b^	+	^209^
	Malignant lymphoma	ND	ND	^7^
Ovary	Ovarian adenocarcinoma	ND	+	^210^
	Clear-cell carcinoma	ND	+	^211^
Pancreas	Pancreas ductal adenocarcinoma	PLIN2, PLIN3, PLIN1	−	^192^
	Clear-cell gastrinoma	ND	+	^212^
Prostate	Prostate gland adenocarcinoma	PLIN2, PLIN3, PLIN1	NC	^192^
	Prostate carcinoma	ND	+	^21,48,50^
Skin	Apocrine-eccrine carcinoma	PLIN2, low PLIN1	+	^213^
	Basal cell skin carcinoma	High PLIN2, low PLIN3	+	^192^
		PLIN2	−	^214^
		Low PLIN2	−	^215^
	Cutaneous apocrine carcinoma	Low PLIN2	ND	^194^
	Cutaneous melanoma	PLIN2	ND	^171^
	Sebaceous adenoma	High PLIN2, PLIN1	−	^192^
		PLIN2	−	^214^
	Sebaceous carcinoma	High PLIN2, PLIN3, PLIN1	+	^192^
		PLIN2	+	^214,215^
		PLIN2, PLIN3, PLIN1	+	^216^
		PLIN1, PLIN2	−	^213^
		High PLIN2	+	^217^
		Low PLIN2, PLIN3	−	^192^
	Skin squamous cell carcinoma	PLIN2	−	^213,214^
		Low PLIN2	+	^215^
Stomach	Gastric adenocarcinoma	PLIN2	+	^189^
		PLIN2	ND	^169,184^
		ND	+	^182^
	Stomach adenocarcinoma	PLIN2, PLIN3, PLIN1	−	^192^
Thyroid	Papillary thyroid carcinoma	High PLIN2	+	^218^

*PLINs* perilipin proteins isoforms, *NC* data presented by the author do not allow for a clear conclusion to be drawn, *ND* not determined.

^a^Protein detection and/or increased (High) or decreased (Low) expression when compared to non tumoral tissue/samples.

^b^Alteration of mRNA levels.

Recent advances in lipidomic detection, particularly in hyperspectral-stimulating Raman scattering microscopy, allowed the evaluation of the individual LD composition in a single cell^177^. This raised the possibility that LD lipid composition could have prognostic value in cancer. Indeed, an aberrant accumulation of CEs in LDs was demonstrated in high-grade and metastatic prostate cancers but not in benign lesions or normal tissues^17,21,50^. Previous data that evaluated the total lipid composition of tumors also showed that CE accumulation was able to differentiate normal from tumor tissue in prostate cancer, leukemia cells, and clear renal cell carcinoma^48,49,178^.

The potential of LDs as a biomarker also came directly from clinical practice in endoscopy for cancer diagnosis. Some works have evaluated whether microvascular patterns on magnifying endoscopy could be used to diagnose benign and malignant lesions, since tumor vessels are structurally and functionally abnormal^179,180^. The presence of a white opaque substance (WOS) that prevented the identification of the microvasculature pattern has been reported^181^ to discriminate benign and malignant lesions. Later, it was shown that the WOS was strongly associated with the presence of LDs, as evidenced by perilipin-2 immunohistochemical and/or Oil red O staining, in gastric, esophageal, and colorectal tumors^182–189^.

## Concluding remarks

Although in the past, the presence of LDs was solely implicated in storage and lipid trafficking, it is currently recognized that these organelles may partake in several cellular functions through a variety of mechanisms. Still, to a great extent, these mechanisms have not yet been fully elucidated. Throughout this review, we discussed features that involve LDs in cancer establishment, pointing out recent evidence that associates these organelles with some of the currently accepted hallmarks of cancer. This draws attention to a potential role of LDs during cancer development, wherein the intricate regulation of LDs could be targeted for drug development or their increased biogenesis inspected as a potential biomarker for diseases. Nevertheless, there are still open questions of fundamental importance to determine a causal relationship between these organelles and tumorigenesis. First, how important is LD biogenesis or lipid metabolism during the distinct phases of tumor development? Second, why is LD accumulation or PAT protein expression in cancer an event that seems to be cell and tissue specific? As discussed above, LDs may have a role during either the initial tumor promotion, by converging mitogenic signaling pathways, partaking in cancer cell metabolism, and providing proinflammatory signaling molecules for TME establishment, or during more advanced cancerous stages, protecting cancer cells from hypoxia and or boosting cells for an aggressive, metastatic phenotype. These points are currently under intense investigation, and therefore, LDs might be suitable candidates for future anticancer therapies.
